# Effect of the Promulgation of the New Migrant’s Employment Law on Migrant Insurance Coverage in Thailand: An Interrupted Time Series Analysis, 2016–2018

**DOI:** 10.3390/ijerph19074384

**Published:** 2022-04-06

**Authors:** Woranan Witthayapipopsakul, Hathairat Kosiyaporn, Sonvanee Uansri, Rapeepong Suphanchaimat

**Affiliations:** 1 International Health Policy Program, Ministry of Public Health, Nonthaburi 11000, Thailand; hathairat@ihpp.thaigov.net (H.K.); sonvanee.u@ihpp.thaigov.net (S.U.); rapeepong@ihpp.thaigov.net (R.S.); 2 Department of Health Services Research and Policy, London School of Hygiene & Tropical Medicine, Faculty of Public Health and Policy, London WC1E 7HT, UK; 3 Bureau of Epidemiology, Department of Disease Control, Ministry of Public Health, Nonthaburi 11000, Thailand

**Keywords:** migrant, interrupted time series, insurance, Thailand

## Abstract

This study explores the effect of the recently enacted Foreigners’ Working Management Emergency Decree, 2017 on migrant insurance coverage between January 2016 and December 2018. We employed an interrupted time series (ITS) model to estimate the level and trend changes of the number of migrants enrolled in Social Health Insurance (SHI) for formal workers and the Health Insurance Card Scheme (HICS) for other migrants. Before the Decree’s implementation, SHI covered roughly a third of the total migrants holding work permits, while HICS covered over half of migrants in the country. We found that the new employment law contributed to a rise in the volume of SHI members and a decline in the HICS members in the long run, which might be partly due to a switch from the HICS members in the formal sector to the SHI, as originally intended by the law. In addition to the law effect, some coincided political force from international trade partners and supranational organizations might also contribute to the progress in protecting the rights of migrant workers. The long-term monitoring of migrant insurance coverage and a mapping against the changes in migrant-related laws and contexts are recommended.

## 1. Background

Human migration has gained significant momentum in policy discussion of the 21st century [1]. Previous literature suggests that migration flow follows the economic theory; in particular, international migrants seek better economic prospects [2]. Together with the growing labor mobility trend, concerns about migrant health have become increasingly discussed as migration policies are not always compatible with the health sector’s goals [1]. The migratory process framework, developed by Zimmerman et al. on migration and health, lays out five phases that policymaking should pay attention to [1]. These are pre-departure, travel, destination, interception, and return phases. These people on the move, especially forced migrants and undocumented workers, are often at risk of deteriorated health due to a lack of access to needed health services in destination countries. In 2015, world leaders pledged their commitments to Universal Health Coverage [3]; however, in many territories, migrants were still left behind, especially when enrolment in health insurance was linked with citizenship or legal migration status [4].

Thailand is a member of the Association of Southeast Asian Nations (ASEAN). It has an estimated population of 66 million. Of its 77 administrative provinces, 33 are border provinces, situated adjacent to Myanmar, Lao PDR, Cambodia, and Malaysia [5]. Thailand has long suffered from an increasing shortage of labor, placing a high demand on migrant workers from its neighboring countries [6,7]. Its higher wage level compared with its neighboring countries have markedly attracted international migrant workers, both legal and illegal [8]. The data of legal migrant workers, provided by the Department of Employment (DOE), Ministry of Labor (MOL), indicate that there has been an increase in the number of the migrant workers, from 1.4 million in 2016 to 2 and 3 million in 2018 and 2019, respectively [9]. The majority of migrants travelled from three neighboring countries [10]. These migrants were mostly engaged in dirty, difficult, and dangerous jobs not attractive to most Thai laborers, such as handicraft, construction, agriculture, and domestic errands [11]. The number of undocumented migrants (those who entered Thailand without valid travel documents and dependants of migrants) was not exactly known.

To work in the country legitimately, the Thai employment law requires a migrant worker to hold a work permit. The process of work permit registration requires a medical check-up alongside the issuance of health insurance [12]. There are two main public insurance schemes for migrant workers: the Social Health Insurance (SHI) and the Health Insurance Card Scheme (HICS) [13]. Enrolment in SHI is restricted to workers who are employed in the formal sector and it is compulsory. The SHI is financed by an equal tripartite contribution from employers, employees, and the Thai government [14]. For migrant workers in the informal sector, the registration process is more complicated. In 2014, the Thai Government launched a nationwide registration policy for all undocumented migrants and their dependants [13]. Once registered with the Government, the formerly undocumented migrants are allowed to be enrolled in the public insurance, namely the HICS, which is managed by the Ministry of Public Health (MOPH). Unlike the SHI, enrolment in the HICS is not mandatory as there is no penalty on the employers if they leave a migrant employee uninsured. The HICS offers various coverage plans that range between THB 365 and THB 4200 (USD 11–132) depending on age and coverage period [13]. The insurance can be purchased at public hospitals in the province where migrants register. The benefits of the HICS and the SSS are similar, covering a vast range of care, from basic treatment to some high-cost interventions [13,15].

Despite the existence of public insurance schemes for migrants, a number of migrants were left uninsured. Previous literature suggests a variety of reasons contributing to the lack of insurance amongst migrants, such as a lack of knowledge and awareness of the insurance package amongst migrants, unaffordability for the less well-off migrants, and red tape in the registration process [16,17,18,19]. To address this, in 2017, the Government exercised a harsh legal measure by promulgating a new law, namely the Foreigners’ Working Management Emergency Decree, B.E. 2560 (2017). The essence of the Decree is to enhance the penalty on both employers of migrants and migrant workers themselves if the employer leaves his/her migrant employees unregistered for a work permit. As the work permit registration came alongside the issuance of health insurance, this implies that the Decree might have an impact on the insurance coverage, particularly the SHI [20].

This study thus aims to evaluate the effect of the new Decree on the insurance coverage for migrant workers in Thailand, using insurance coverage data between January 2016 and December 2018.

## 2. Methods

### 2.1. Study Design

We conducted an interrupted time series (ITS) analysis on time series cross-sectional data to estimate the level and trend change in the number of migrants enrolled in the SHI and the HICS. This method is a useful tool, which is commonly exercised for evaluating policy interventions and estimating the change in trend following the intervention of interest [21,22,23].

### 2.2. Data Sources

Three sets of data were used. First, we obtained monthly statistics of migrants holding work permits from the Foreign Workers Administration Office of the DOE, which was a publicly accessible dataset [24]. Second, we retrieved the number of migrant workers insured with the SHI from the Social Security Office (SSO), the MOL. Last, we contacted the MOPH to access monthly numbers of HICS membership. All data were obtained in an aggregated monthly format. We found that the most complete datasets for the three sources were between January 2016 and December 2018. We therefore divided the data into two equal intervals (before and after the intervention). The Decree was introduced in month 19 (setting January 2016 as month 1).

### 2.3. Analysis

We estimated SHI and HICS coverage by dividing the numbers of SHI and HICS members with all documented migrant workers (migrants holding a work permit) before inputting these data into the model. The analysis followed a multiple linear regression, $Y_{t}\;=\;\beta_{0}\;+\;\beta_{1}\;*\;time\;+\;\beta_{2}\;*\;intervention\;+\;\beta_{3}\;*\;postslope\;+\;\varepsilon_{t}$, where $Y_{t}$ was the dependent variable (insurance coverage) at time t; t indicated the time variable during the observation period; intervention was coded 0 for months 1–18 and coded 1 for months 19 onwards. $\beta_{0}$ represented the baseline coverage at the start of study period $Y_{0}$; $\beta_{1}$ estimated the baseline trend irrespective of intervention effect (effect of time trend); $\beta_{2}$ reflected the change in level estimated immediately after the intervention; $\beta_{3}$ described the trend change of the migrant insurance registration after the introduction of the intervention; and $\varepsilon_{t}$ denoted the error term. The counterfactual situation was estimated by the following equation: $Y_{t}\;=\;\beta_{0}\;+\;\beta_{1}\;*\;time\;+\;\varepsilon_{t}$. We controlled for autocorrelation in the data series using a generalized least squares estimator, Prais–Winsten, and Cochrane–Orcutt regression. We later used the coefficient from the regression to construct a counterfactual graph, representing what would have occurred if the Decree had not been introduced—then, we compared the counterfactual number with the actual figure of migrant enrollees for both insurance schemes.

To understand the effect of law implementation across geography, the results were presented for three different regions: (i) nationwide dataset (77 provinces), (ii) Greater Bangkok (Bangkok and its five surrounding provinces where industrial estates condense), and (iii) 33 border provinces where most migrants were engaged in the informal sector, such as agriculture business and informal construction. We used Stata/SE 16.1 (Serial Number 401609332499) to perform all statistical computations.

### 2.4. Ethics Consideration

As this study was part of the monitoring of the health system performance of the International Health Policy Program, the MOPH, and we used only secondary data accessible to the public, no ethics clearance was required. However, we followed the ethical standards for research by not exposing the individual information in the datasets and thus all personal information was kept anonymous.

## 3. Results

The descriptive statistics about all documented migrant workers and those enrolled in the SHI and the HICS are presented in Table 1. Over the period of three years, Thailand saw a steady increase in the monthly average of the number of work permit holders, rising from 1.51 million in 2016 to 2.23 million in 2018. Mean and median figures did not show a marked difference. Greater Bangkok shared around 50–56% and the 33 border provinces accounted for 18–23% of the total figures. The 3-year trends in these two geographical regions also followed the country trend. The numbers of SHI and HICS members combined exceeded the numbers of all work permit holders for certain months.

Overall, we found that the number of HICS enrollees remained higher than the number of SHI enrollees throughout the observed period, but the gap became much smaller in 2018, when the numbers of HICS dropped and SHI rose. From the macro-perspective of all 77 provinces in Thailand, the number of migrants insured with the HICS remained stable at approximately 1.5–1.6 million in 2016–2017, and then dropped to approximately 1.1–1.3 million in 2018.

The coverage of SHI membership remained at around a third of all work permit holders in 2016–2017 but peaked to nearly a half in 2018. A similar picture was observed for Greater Bangkok. In contrast, the HIC enrollees exceeded the number of all work permit holders (109–132%) and remained around 3–5 times larger than the SHI enrollees in the border provinces during these years.

Table 2 presents the effect of the Foreigners’ Working Management Emergency Decree, B.E. 2560 (2017) on insurance coverage from Prais-Winsten and Cochrane-Orcutt regression. The SHI coverage had been, on average, 33.92% for the whole country; 38.36% in Greater Bangkok; and 22.86% in border provinces. The data did not support that there was a significant month-to-month change for all geographical areas. After the Decree was implemented, there was no significant change observed for all provinces and Greater Bangkok, but the SHI coverage showed an immediate drop by 9.75% in the border provinces. The monthly enhancement after the implementation of the law, however, appeared to be 1.86 for the whole of Thailand; 1.67 for Greater Bangkok; and 1.77 for the 33 provinces.

For the HICS, the baseline coverage was 82.93% for the whole country, 58.73% for Greater Bangkok, and 87.34% for the border provinces. The increasing baseline monthly coverage trend was observed by 1.47%, 1.40%, and 3.14%. No significant change was expected immediately after the Decree’s promulgation. However, a negative trend change in the monthly coverage by 5.11–5.68% was observed in all areas, with statistical significance when assessed against a *p*-value cut-off at 0.05.

**Figure 1:**
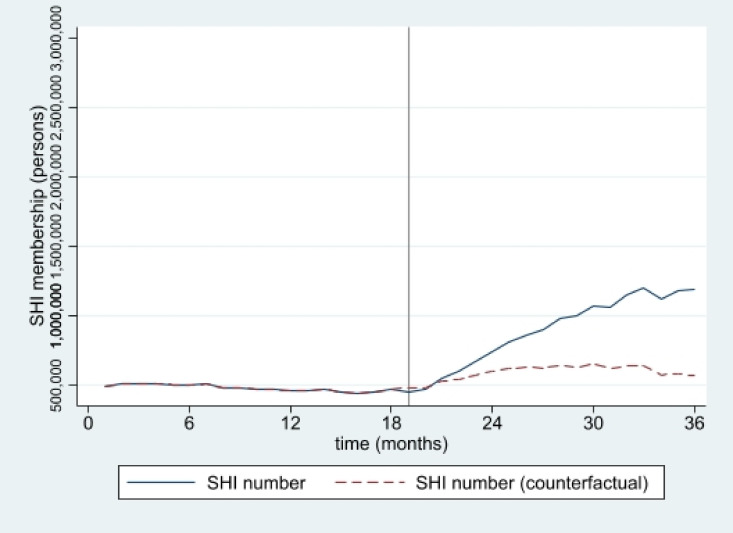
SHI membership, all provinces (2016–2018).

**Figure 2:**
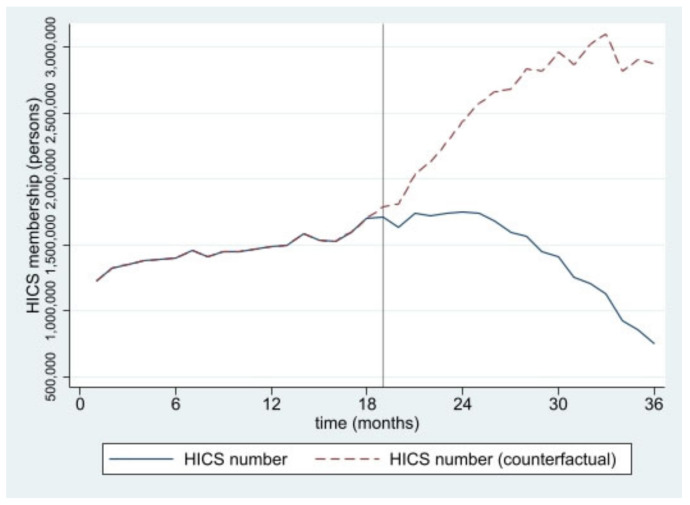
HICS membership, all provinces (2016–2018).

**Figure 3:**
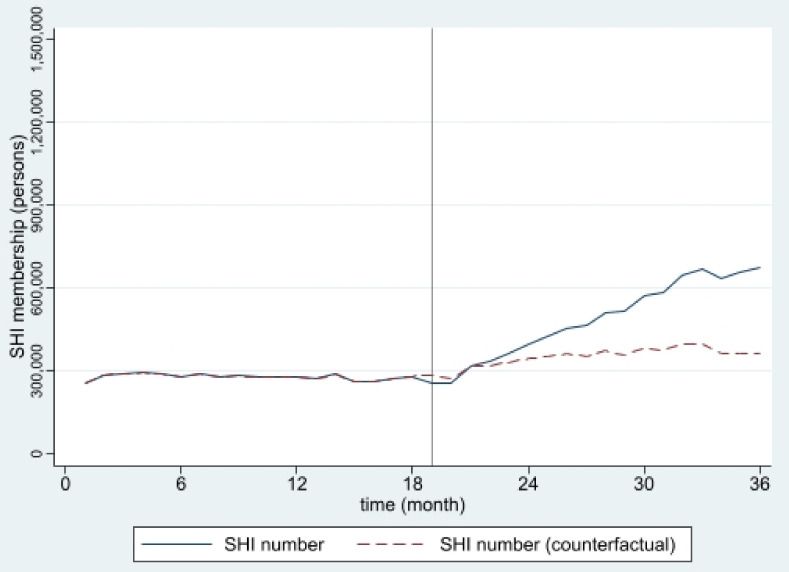
SHI membership, Greater Bangkok (2016–2018).

**Figure 4:**
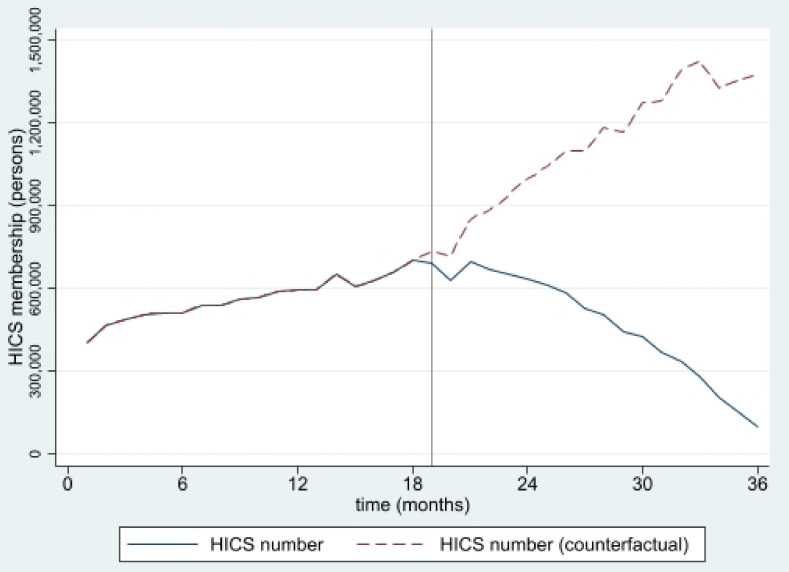
HICS membership, Greater Bangkok (2016–2018).

**Figure 5:**
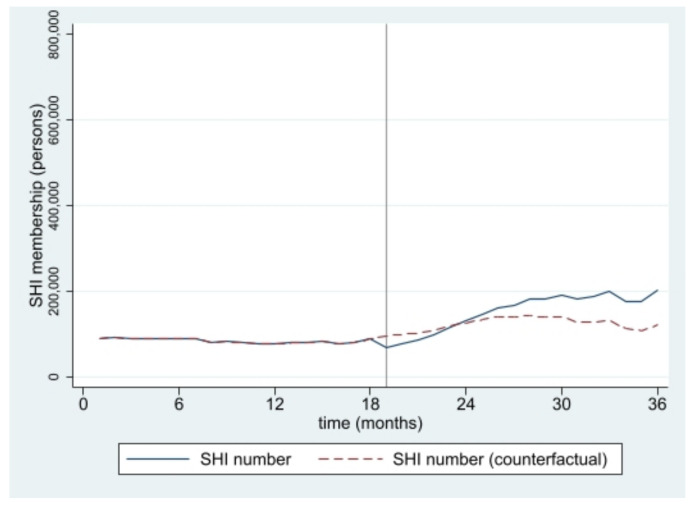
SHI membership, border provinces (2016–2018).

**Figure 6:**
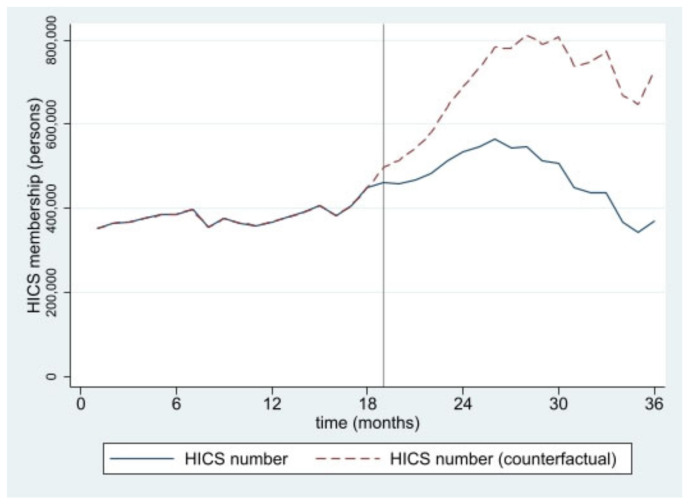
HICS membership, border provinces (2016–2018).

The estimated numbers of SHI and HICS during the 36 months from January 2016 to December 2018 from interrupted time series models are pictorially presented in Figure 1, Figure 2, Figure 3, Figure 4, Figure 5 and Figure 6. In Figure 1 and Figure 2, showing the results of Thailand as a whole, the SHI enrollees plateaued at around 0.5 million before the intervention and sharply rose to 1.2 million at the end of the observed period, approximately twice the counterfactual estimate. On the contrary, a slightly rising trend of the HICS members was observed (approximately 1.2–1.7 million), but they gradually dropped to 0.8 million by month 36 (Figure 2). This pattern showed a marked difference from the expected trend had the Decree never been in place. As shown in Figure 3, Greater Bangkok saw a similar picture with SHI membership, staying at approximately 0.3 million before the implementation and rising sharply to nearly 0.7 million in December 2018 (while the counterfactual number was expected to reach a peak at around 0.4 million). The HICS members climbed from 0.4 to 0.7 million before dropping down to around 0.2 million, roughly 15% of the counterfactual estimate, as shown in Figure 4. Lastly, for the border provinces, Figure 5 presents the SHI membership trend, which was similar to that of the whole country and Greater Bangkok but slightly more fluctuating. Before the intervention, SHI contained approximately 70,000–90,000 members, but after the Decree was launched, the number grew roughly threefold (180,000–200,000) by the end of the study period. The counterfactual SHI number also followed the same pattern but with smaller magnitude (varying between 120,000 and 150,000). For the HICS (Figure 6), the enrollee toll was estimated to reach a peak at around 800,000 by month 27 (counterfactual), but in reality, the peak was much smaller, at around 550,000, and then the trend dropped steadily to the baseline level (around 350,000) by the last observed month.

## 4. Discussion

Overall, this study is amongst the first studies to apply rigorous statistical techniques to evaluate the new law on the employment of migrants in Thailand. Moreover, it is amongst the very few studies in the migrant field that highlight the relationship among the employment law and the health security of migrants, which is a significant concern in not only Thailand but also many other nations where migrant health is in the political spotlight. We found that the new employment law implemented in 2017 was associated with a decline in HICS members in the long run but, at the same time, contributed to a rise in the volume of SHI members, though an immediate change in the insurance enrollment was not obvious. A possible explanation is that the Decree caused a switch from the HICS members in the formal sector to the SHI as intended by the law. This phenomenon reflects some long-standing operational problems of the SHI enrolment, which has resulted in migrants enrolling in the more convenient HICS instead. First, the registration of the HICS is easier for employers than that of the SHI. As the HICS targets migrant workers in the informal sector, where an employer is not required to register his/her business with the MOL, the documents required for purchasing the insurance are only the residence permit of the purchaser and the name and address of the employer. Second, the HICS is solely governed by the MOPH. It does not have a strong legal instrument to support its enforcement—unlike the SHI, which is founded by the employment law [25]. Last, recent evidence showed that most migrants preferred the HICS over the SHI as the payment system for the HICS is based on an annual payment regardless of employment status and employers, as opposed to a monthly deduction of the insured’s salary required by the SHI [26].

Another interesting finding is the discovery that the SHI and HICS combined outnumbered the work permit holders. This reflected the non-synchroneity of the data recording systems between the MOPH and the MOL. Migrants returning to their home countries (thus dropping out from the work permit holder list) might still have their names existing in the insured list (especially the HICS, which is regulated by the MOPH and not the MOL). Another possible explanation is the duplication in the name lists of the HICS and the SHI as there is a three-month transition period during which the insured need to have their salary deducted for three months before the SHI is activated. The applicants are encouraged to buy the HICS during this period. It is still possible that the applicant paid with the full annual cost to have full one-year coverage [10].

We also found that the number of HICS was relatively high in the border provinces, where nature of the economy mostly relies on informal businesses, including seasonal cropping or informal construction [6,7,27].

It should be noted that the observed increasing SHI coverage might not be the sole effect of the 2017 Decree. There are also coincided political force and international events that might lead to more migrants earning formal employment status. Thailand has been pressured by its important trade partners, notably the United States (US) and the European Union (EU), on human trafficking, slavery, and illegal unreported unregulated (IUU) fishing [28,29]. In 2015, the EU issued a yellow card to Thailand, threatening to ban its seafood exports unless significant progress was made to fight against IUU fishing in its waters [30]. At the same time, the US has watched Thailand closely on actions regarding human trafficking and labor rights using the trade benefits’ Generalized System of Preferences (GSP) as an economic sanction tool. These important threats forced the country to make some rapid progress in protecting the health of migrants [28]. A concrete movement was the ratification of many international agreements that aim to ensure better living standards for migrants, including the 1930 Forced Labor Convention and the 2007 Work in Fishing Convention [31], and the 2017 ASEAN Declaration on the Protection and Promotion of the Rights of Migrant Workers [32]. This coincides with a study by Herbenholz which suggested that the external political force and the concern of reputational effects in foreign affairs emerged as a very powerful tool to influence the priority setting of migrant policies in Thailand [33]. Feldbaum et al. underpin that health has long been intertwined with the foreign policies of states, especially in aid, trade, diplomacy, and national security [34].

From a macro-view, the Decree is successful in expanding the health security of the migrants through SHI. However, the SHI has not cleared up all underlying problems concerning the insurance coverage for migrants. For instance, the SHI protection stops if either the employer or employee stops paying a monthly contribution for at least three consecutive months [35]. More importantly, it does not cover the workers’ dependants. Though the HICS provides room for covering the workers’ dependants, its voluntary nature means that full coverage is far from achievable because of the adverse selection issue, and there are also concerns about financial unsustainability [36]. It is estimated that the volume of non-registered migrant workers and their dependants combined amounts to over two million [7,35].

To tackle this problem, certain provinces along the Thai-Myanmar border have implemented their own initiatives. Some worked closely with a private not-for-profit organization to initiate a voluntary micro-insurance program. The nature of such initiatives is quite similar; that is, by allowing those dropping out from the SHI and the HICS, or non-registered migrants and migrant children, to be insured with the insurance scheme internally created by the provinces [18,27]. However, these initiatives always suffer from adverse selection due to inadequate risk pooling—the same phenomenon faced elsewhere abroad [37,38]. A more desirable financing approach applied in the European Union, which allows migrants eligible for health coverage in one member country to access healthcare in other member countries, is difficult to replicate in other parts of the world. One of the common arguments for not including migrants is a fear of increased health expenditure, although there are several valid counter-arguments that covering migrants have actually resulted in cost savings and economic benefits in the longer term, as supported by studies in Malaysia [39], Germany [40,41], and some other European countries [42]. Despite the ASEAN platform, such cross-border agreement would require a series of high-level negotiations, and they would need to consider substantial differences in cost and health system maturity between origin and destination countries [4,43]. A review in 2015 focusing on migrant health insurance policies in five ASEAN countries revealed that they were at varying stages: Thailand allowed migrants to opt into public insurance schemes, while Malaysia and Singapore were yet to consider migrants’ inclusion in their state-run UHC system as recipient countries; the Philippines provided some limited insurance benefits to its outbound migrants [44].

Our study provides an example of an intervention that positively affects migrant health protection in a middle-income country setting and describes related challenges that may be useful for other countries with similar context. By showing research evidence on the impact of a non-health policy, our study advocates for better policy coherence across sectors and good policy governance in the near future. This study is not without limitations. The major weakness is the lack of undocumented migrant data, which are extremely difficult to trace. This limitation inevitably compromises the accuracy of the insurance coverage. In addition, the HICS dataset available to us is not granular enough to sort migrants by work status, such as how many are currently engaged in the informal sector and how many formal-sector migrants were insured with the HICS. Moreover, the results should be cautiously interpreted as migrant health policy is extremely dynamic. The impact of the Decree is always subject to change given new policies or changes in the contextual environment, and international policies that affect migrant flow, such as the fluctuations in the country’s economy, will definitely have an impact.

Further studies that explore the factors influencing the enrolment of the different insurance schemes at an individual level are recommended, including research on proper and feasible insurance options for non-Thais as a whole. The existing insurance schemes with a larger financing pool (such as those already operated for Thai citizens) should also be considered to accommodate migrants. An approach that unbinds the employment process and insurance entitlement should be contemplated. Further studies that investigate factors associated with the enrolment of different insurance schemes at an individual level and a mapping of migrant insurance coverage against the changes in migrant-related laws and contexts are recommended.

## 5. Conclusions

The new employment law on migrants in Thailand, proclaimed in 2017, contributed to a positive change in SHI enrolment. In contrast, the volume of HICS insurees gradually declined after the introduction of the law. The rising trend of the SHI insuree toll might not be due to the new law alone, but also a result of the political context and foreign affairs influence at that time. Continued actions to promote migrants’ health security should be maintained, with special attention given to undocumented migrants and migrants’ dependants. Further research should explore proper insurance options, especially a scheme with a larger financing pool for all non-Thai residents. A mapping of migrant insurance coverage against changes in migrant-related laws and the contextual environment of the employment policies for migrants would be useful.

## Figures and Tables

**Table 1:** Average numbers of migrant insurance coverage, 2016–2018.

Years		Social Health Insurance (SHI)—*n*	Health Insurance Card Scheme (HICS)—*n*	Work Permit Holders—*n*
**All provinces**				
2016	Mean (sd)	488,305 (24,386)	1,443,654 (199,518)	1,514,443 (36,762)
	Median (min–max)	492,963 (424,622–514,471)	1,510,908 (1,141,715–1,683,121)	1,519,111 (1,451,717–1,564,106)
2017	Mean (sd)	528,816 (49,458)	1,614,758 (177,228)	1,652,313 (210,673)
	Median (min–max)	502,504 (485,864–633,513)	1,502,834 (1,467,533–1,877,236)	1,585,838 (1,437,716–2,062,807)
2018	Mean (sd)	1,027,702 (233,819)	1,290,153 (480,744)	2,227,335 (84,008)
	Median (min–max)	1,162,366 (651,834–1,216,231)	1,153,788 (822,781–2,121,411)	2,214,999 (2,119,413–2,356,454)
**Greater Bangkok**				
2016	Mean (sd)	275,410 (15,844)	543,256 (117,572)	769,309 (34,164)
	Median (min–max)	277,737 (231,958–290,615)	592,282 (377,083–659,449)	780,842 (666,687–793,718)
2017	Mean (sd)	301,657 (30,223)	640,520 (54,094)	877,587 (109,296)
	Median (min–max)	287,023 (263,577–363,555)	615,404 (580,743–726,280)	834,747 (763,379–1,079,125)
2018	Mean (sd)	565,357 (121,561)	362,223 (241,832)	1,230,196 (76,507)
	Median (min–max)	622,012 (370,924–677,954)	279,344 (155,357–772,912)	1,250,200 (1,113,123–1,356,655)
**Border provinces**				
2016	Mean (sd)	86,489 (10,818)	379,672 (43,726)	347,666 (37,252)
	Median (min–max)	90,144 (53,131–92,248)	378,498 (325,252–450,625)	363,108 (294,262–389,708)
2017	Mean (sd)	88,461 (12,113)	439,736 (76,892)	334,093 (46,808)
	Median (min–max)	86,004 (61,428–108,348)	396,432 (368,933–552,549)	325,852 (277,255–423,766)
2018	Mean (sd)	175,561 (37,370)	465,369 (93,745)	412,794 (47,121)
	Median (min–max)	194,803 (114,406–207,922)	436,162 (368,504–636,288)	423,846 (328,286–464,177)

**Table 2:** Estimates of migrant insurance coverage from Prais–Winsten and Cochrane–Orcutt regression.

	All Provinces		Bangkok and Vicinity		Border Provinces	
	Coefficient (95% CI)	*p*-Value	Coefficient (95% CI)	*p*-Value	Coefficient (95% CI)	*p*-Value
**Social Health Insurance (SHI)**						
Constant$\;\beta_{0}$	33.92 (26.83, 41.01)	<0.001	38.36 (33.30, 43.42)	<0.001	22.86 (15.58, 30.15)	<0.001
Time	−0.20 (−0.80, 0.41)	0.511	−0.27 (−0.72, 0.19)	0.239	0.29 (0.35, 0.94)	0.361
Intervention	−4.16 (−10.63, 2.30)	0.199	−5.03 (−11.19, 1.12)	0.105	−9.75 (−18.02, −1.48)	0.022
Postslope	1.86 (0.88, 2.83)	0.001	1.67 (0.99, 2.35)	<0.001	1.77 (0.78, 2.76)	0.001
**Health Insurance Card Scheme (HICS)**						
Constant$\;\beta_{0}$	82.93 (66.38, 99.48)	<0.001	58.73 (42.10, 75.37)	<0.001	87.34 (68.38, 106.30)	<0.001
Time	1.47 (0.03, 2.90)	0.045	1.40 (−0.02, 2.83)	0.053	3.14 (1.46, 4.82)	0.001
Intervention	0.48 (−16.31, 17.27)	0.954	0.77 (−14.90, 16.44)	0.921	−6.10 (−27.59, 15.39)	0.567
Postslope	−5.59 (−7.87, −3.32)	<0.001	−5.68 (−7.97, −3.38)	<0.001	−5.11 (−7.69, −2.53)	<0.001

## Data Availability

Monthly statistics of migrants holding work permit from the Foreign Workers Administration Office can be accessed at https://www.doe.go.th/prd/alien/statistic/param/site/152/cat/82/sub/0/pull/category/view/list-label, accessed on 1 April 2022 (Thai language only). The statistics of SHI and HICS enrollment can be requested directly to the Social Security Office and the Ministry of Public Health, Thailand.
